# 1-Hy­droxy­isoquinolin-2-ium hydrogen succinate

**DOI:** 10.1107/S1600536814006485

**Published:** 2014-03-29

**Authors:** S. Ambalatharasu, A. Sankar, G. Peramaiyan, G. Chakkaravarthi, R. Kanagadurai

**Affiliations:** a Department of Physics, Presidency College, Chennai 600 005, India; b Department of Physics, CPCL Polytechnic College, Chennai 600 068, India

## Abstract

In the title salt, C_9_H_8_NO^+^·C_4_H_5_O_4_^−^, the isoquinolinium ring system is approximately planar [r.m.s deviation = 0.011 (2) Å]. In the crystal, adjacent cations and anions are linked by O—H⋯O and N—H⋯O hydrogen bonds, forming columns along the *b* axis. The columns are connected by weak C—H⋯O inter­actions into a three-dimensional network.

## Related literature   

For the biological activity of quinoline derivatives, see: Hopkins *et al.* (2005 ▶); Musiol *et al.* (2006 ▶). For bond-length data, see: Allen *et al.* (1987 ▶). For a related quinoline structure, see: Loh *et al.* (2010 ▶).
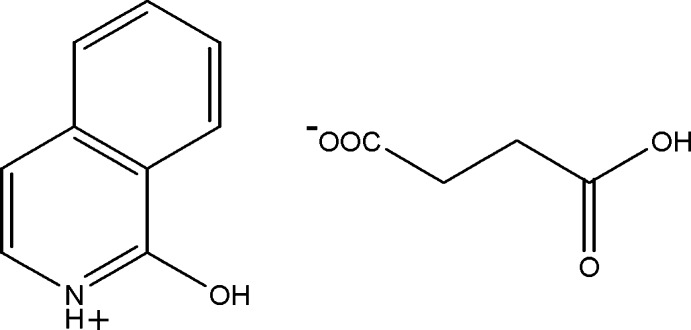


## Experimental   

### 

#### Crystal data  

C_9_H_8_NO^+^·C_4_H_5_O_4_^−^
*M* *_r_* = 263.24
Monoclinic, 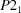

*a* = 9.553 (5) Å
*b* = 4.962 (3) Å
*c* = 12.706 (5) Å
β = 104.117 (5)°
*V* = 584.1 (5) Å^3^
*Z* = 2
Mo *K*α radiation
μ = 0.12 mm^−1^
*T* = 295 K
0.22 × 0.18 × 0.16 mm

#### Data collection  

Bruker Kappa APEXII CCD diffractometer
Absorption correction: multi-scan (*SADABS*; Sheldrick, 1996 ▶) *T*_min_ = 0.975, *T*_max_ = 0.982
8297 measured reflections
3688 independent reflections
3289 reflections with *I* > 2σ(*I*)
*R*_int_ = 0.028

#### Refinement  

*R*[*F* ^2^ > 2σ(*F* ^2^)] = 0.053
*wR*(*F* ^2^) = 0.169
*S* = 1.10
3688 reflections
181 parameters
4 restraints
H atoms treated by a mixture of independent and constrained refinement
Δρ_max_ = 0.57 e Å^−3^
Δρ_min_ = −0.55 e Å^−3^

### 

Data collection: *APEX2* (Bruker, 2004 ▶); cell refinement: *SAINT* (Bruker, 2004 ▶); data reduction: *SAINT*; program(s) used to solve structure: *SHELXS97* (Sheldrick, 2008 ▶); program(s) used to refine structure: *SHELXL97* (Sheldrick, 2008 ▶); molecular graphics: *PLATON* (Spek, 2009 ▶); software used to prepare material for publication: *SHELXL97*.

## Supplementary Material

Crystal structure: contains datablock(s) global, I. DOI: 10.1107/S1600536814006485/is5349sup1.cif

Structure factors: contains datablock(s) I. DOI: 10.1107/S1600536814006485/is5349Isup2.hkl

Supporting information file. DOI: 10.1107/S1600536814006485/is5349Isup3.cml

CCDC reference: 993415

Additional supporting information:  crystallographic information; 3D view; checkCIF report

## Figures and Tables

**Table 1:** Hydrogen-bond geometry (Å, °)

*D*—H⋯*A*	*D*—H	H⋯*A*	*D*⋯*A*	*D*—H⋯*A*
N1—H1⋯O3^i^	0.92 (1)	2.48 (2)	3.255 (3)	142 (3)
O1—H1*A*⋯O4^ii^	0.81 (1)	1.91 (1)	2.705 (2)	170 (3)
O2—H2*A*⋯O5^iii^	0.83 (1)	1.77 (1)	2.591 (2)	169 (3)
C4—H4⋯O3^iv^	0.93	2.50	3.360 (3)	154
C8—H8⋯O5^v^	0.93	2.34	3.078 (3)	137
C9—H9⋯O4^v^	0.93	1.81	2.718 (2)	165

Symmetry codes: (i) 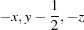; (ii) 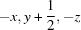; (iii) 
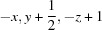
; (iv) 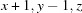; (v) 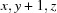.

**Fig. 1.**
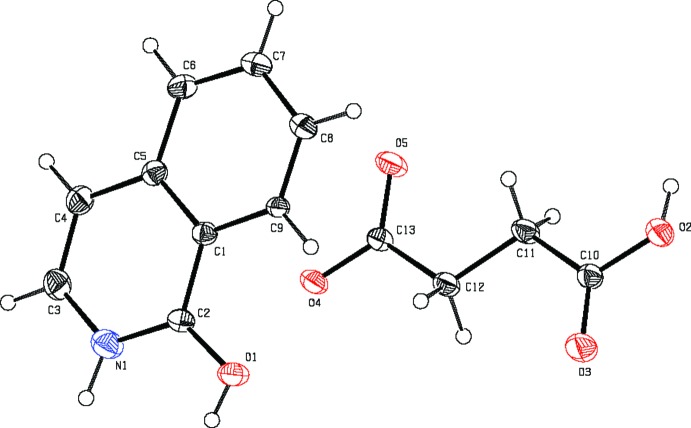
The molecular structure of the title cpompound, with atom labels and 30% probability displacement ellipsoids for non-H atoms.

**Fig. 2.**
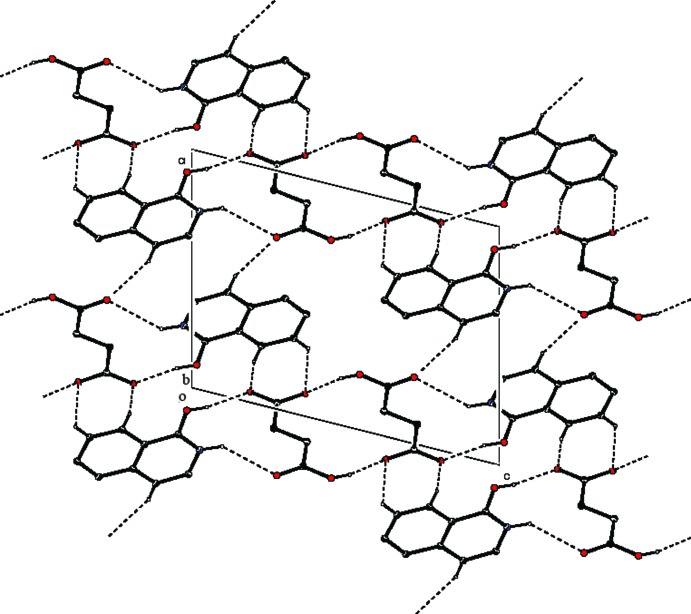
The packing diagram of the title compound, viewed down the b axis. Intermolecular hydrogen bonds are shown as dashed lines. H atoms not involved in hydrogen bonding have been omitted.

## supplementary crystallographic information

### 1. Comment

Quinolinium derivatives are known to exhibit interesting bioactivities and pharmacological activities (Hopkins *et al.*, 2005; Musiol *et al.*, 2006). We herewith report the crystal structure of the title compound (Fig. 1). The bond lengths of the anion are within normal range (Allen *et al.*, 1987) and the bond lengths of cation are comparable with the reported similar structure (Loh *et al.*, 2010).

In the cation, the isoquinolinium ring system is planar [r.m.s deviation = 0.011 (2) Å]. The adjacent cations and anions are linked by weak intermolecular O—H···O and N—H···O interactions (Table 1 and Fig. 2 ) to form a column along the *b* axis. Weak π–π [*Cg*1···*Cg*1 (1-x, 1/2+y, -z) distance = 4.994 (3) Å, *Cg*1···*Cg*2 (x, -1+y, z) distance = 4.673 (3) Å; *Cg*1 and *Cg*2 are the centroids of the rings (N1/C1–C5) and (C1/C5–C9), respectively] and C—H···O interactions are also observed in the crystal structure.

### 2. Experimental

1-Hydroxyisoquinolin-2-ium succinate was synthesized using the raw materials 1-hydroxyisoquinoline (1.45 g) and succinic acid (1.18 g) in an equimolar ratio. These reactants were dissolved in 10 ml of ethanol solvent and yellow precipitate was obtained after some time. The precipitate was dissolved in the same solvent and it is kept at room temperature for crystallization. After a span of four days, rod like crystals for diffraction study were harvested.

### 3. Refinement

H atoms for C_aromatic_H and CH_2_ were positioned geometrically and refined using riding model, with C—H = 0.93 and 0.97 Å, respectively, and with *U*_iso_(H) = 1.2*U*_eq_(C). H atoms bounded to N and O atoms were located in a difference Fourier map and refined with *U*_iso_(H) = 1.5*U*_eq_(N, O) and distance restraints of O—H = 0.82 (1) Å and N—H = 0.86 (1) Å. One reflection (0 0 1) was omited during refinement as it was showing poor agreement.

### Crystal data

**Table:**

C_9_H_8_NO^+^·C_4_H_5_O_4_^−^	*F*(000) = 276
*M**_r_* = 263.24	*D*_x_ = 1.497 Mg m^−^^3^
Monoclinic, *P*2_1_	Mo *K*α radiation, λ = 0.71073 Å
Hall symbol: P 2yb	Cell parameters from 4664 reflections
*a* = 9.553 (5) Å	θ = 2.2–32.6°
*b* = 4.962 (3) Å	µ = 0.12 mm^−^^1^
*c* = 12.706 (5) Å	*T* = 295 K
β = 104.117 (5)°	Block, colourless
*V* = 584.1 (5) Å^3^	0.22 × 0.18 × 0.16 mm
*Z* = 2	

### Data collection

**Table:**

Bruker Kappa APEXII CCD diffractometer	3688 independent reflections
Radiation source: fine-focus sealed tube	3289 reflections with *I* > 2σ(*I*)
Graphite monochromator	*R*_int_ = 0.028
ω and φ scan	θ_max_ = 33.2°, θ_min_ = 2.2°
Absorption correction: multi-scan (*SADABS*; Sheldrick, 1996)	*h* = −13→14
*T*_min_ = 0.975, *T*_max_ = 0.982	*k* = −6→7
8297 measured reflections	*l* = −19→19

### Refinement

**Table:**

Refinement on *F*^2^	Primary atom site location: structure-invariant direct methods
Least-squares matrix: full	Secondary atom site location: difference Fourier map
*R*[*F*^2^ > 2σ(*F*^2^)] = 0.053	Hydrogen site location: inferred from neighbouring sites
*wR*(*F*^2^) = 0.169	H atoms treated by a mixture of independent and constrained refinement
*S* = 1.10	*w* = 1/[σ^2^(*F*_o_^2^) + (0.1138*P*)^2^ + 0.0584*P*] where *P* = (*F*_o_^2^ + 2*F*_c_^2^)/3
3688 reflections	(Δ/σ)_max_ < 0.001
181 parameters	Δρ_max_ = 0.57 e Å^−^^3^
4 restraints	Δρ_min_ = −0.55 e Å^−^^3^

### Special details

**Table:**

Geometry. All esds (except the esd in the dihedral angle between two l.s. planes) are estimated using the full covariance matrix. The cell esds are taken into account individually in the estimation of esds in distances, angles and torsion angles; correlations between esds in cell parameters are only used when they are defined by crystal symmetry. An approximate (isotropic) treatment of cell esds is used for estimating esds involving l.s. planes.
Refinement. Refinement of F^2^ against ALL reflections. The weighted R-factor wR and goodness of fit S are based on F^2^, conventional R-factors R are based on F, with F set to zero for negative F^2^. The threshold expression of F^2^ > 2sigma(F^2^) is used only for calculating R-factors(gt) etc. and is not relevant to the choice of reflections for refinement. R-factors based on F^2^ are statistically about twice as large as those based on F, and R- factors based on ALL data will be even larger.

### Fractional atomic coordinates and isotropic or equivalent isotropic displacement parameters (Å^2^)

**Table:**

	*x*	*y*	*z*	*U*_iso_*/*U*_eq_	
C1	0.28708 (17)	1.0029 (4)	0.15427 (13)	0.0248 (3)	
C2	0.21024 (18)	1.0069 (4)	0.04383 (12)	0.0268 (3)	
C3	0.3651 (2)	0.6470 (5)	0.01057 (17)	0.0378 (4)	
H3	0.3901	0.5288	−0.0387	0.045*	
C4	0.4391 (2)	0.6380 (4)	0.11717 (17)	0.0345 (4)	
H4	0.5135	0.5146	0.1404	0.041*	
C5	0.40134 (18)	0.8183 (4)	0.19153 (14)	0.0273 (3)	
C6	0.4726 (2)	0.8221 (5)	0.30338 (15)	0.0352 (4)	
H6	0.5492	0.7052	0.3300	0.042*	
C7	0.4292 (2)	0.9972 (5)	0.37231 (14)	0.0381 (4)	
H7	0.4749	0.9978	0.4459	0.046*	
C8	0.3161 (2)	1.1745 (5)	0.33147 (14)	0.0342 (4)	
H8	0.2870	1.2942	0.3783	0.041*	
C9	0.24902 (16)	1.1760 (3)	0.22686 (11)	0.0219 (3)	
H9	0.1742	1.2977	0.2021	0.026*	
C10	−0.2126 (2)	1.2254 (4)	0.36224 (13)	0.0294 (4)	
C11	−0.1045 (2)	0.9998 (5)	0.37431 (13)	0.0337 (4)	
H11A	−0.1356	0.8529	0.4136	0.040*	
H11B	−0.0122	1.0633	0.4174	0.040*	
C12	−0.0841 (2)	0.8927 (4)	0.26675 (13)	0.0303 (3)	
H12A	−0.1760	0.8251	0.2246	0.036*	
H12B	−0.0559	1.0410	0.2266	0.036*	
C13	0.02711 (18)	0.6711 (4)	0.27745 (12)	0.0258 (3)	
N1	0.2522 (2)	0.8310 (5)	−0.02600 (14)	0.0423 (4)	
H1	0.214 (3)	0.848 (9)	−0.0992 (9)	0.063*	
O1	0.10057 (16)	1.1826 (3)	0.01569 (11)	0.0364 (3)	
H1A	0.067 (3)	1.149 (7)	−0.0474 (11)	0.055*	
O2	−0.23278 (17)	1.3291 (4)	0.45355 (11)	0.0410 (4)	
H2A	−0.198 (3)	1.257 (7)	0.5134 (15)	0.061*	
O3	−0.27879 (19)	1.3135 (5)	0.27647 (12)	0.0492 (5)	
O4	0.04477 (15)	0.5692 (3)	0.19024 (9)	0.0332 (3)	
O5	0.09610 (18)	0.5944 (4)	0.36893 (10)	0.0425 (4)	

### Atomic displacement parameters (Å^2^)

**Table:**

	*U*^11^	*U*^22^	*U*^33^	*U*^12^	*U*^13^	*U*^23^
C1	0.0245 (7)	0.0250 (7)	0.0242 (6)	−0.0023 (6)	0.0042 (5)	0.0003 (6)
C2	0.0285 (8)	0.0263 (8)	0.0234 (6)	−0.0023 (7)	0.0023 (5)	−0.0009 (6)
C3	0.0405 (10)	0.0367 (10)	0.0375 (9)	0.0019 (8)	0.0118 (7)	−0.0097 (8)
C4	0.0335 (8)	0.0329 (10)	0.0371 (9)	0.0060 (7)	0.0088 (7)	0.0011 (7)
C5	0.0259 (7)	0.0275 (8)	0.0284 (6)	−0.0007 (7)	0.0064 (6)	0.0019 (6)
C6	0.0308 (8)	0.0414 (11)	0.0305 (7)	0.0065 (8)	0.0015 (6)	0.0067 (7)
C7	0.0390 (10)	0.0474 (12)	0.0246 (7)	0.0031 (9)	0.0010 (6)	0.0000 (8)
C8	0.0363 (9)	0.0406 (10)	0.0244 (7)	0.0025 (8)	0.0048 (6)	−0.0041 (7)
C9	0.0215 (6)	0.0236 (7)	0.0190 (5)	0.0008 (6)	0.0022 (5)	−0.0010 (5)
C10	0.0318 (8)	0.0310 (9)	0.0251 (6)	0.0048 (7)	0.0065 (6)	0.0020 (6)
C11	0.0425 (10)	0.0346 (9)	0.0219 (6)	0.0133 (8)	0.0038 (6)	0.0019 (6)
C12	0.0359 (9)	0.0322 (9)	0.0215 (6)	0.0067 (7)	0.0047 (6)	0.0013 (6)
C13	0.0290 (7)	0.0249 (8)	0.0220 (6)	0.0003 (6)	0.0036 (5)	−0.0006 (5)
N1	0.0468 (10)	0.0450 (11)	0.0331 (7)	−0.0002 (9)	0.0058 (7)	−0.0062 (8)
O1	0.0384 (7)	0.0378 (8)	0.0273 (6)	0.0087 (6)	−0.0031 (5)	−0.0047 (5)
O2	0.0439 (8)	0.0501 (9)	0.0283 (6)	0.0138 (7)	0.0077 (5)	−0.0033 (6)
O3	0.0540 (9)	0.0608 (12)	0.0310 (6)	0.0270 (9)	0.0069 (6)	0.0116 (7)
O4	0.0389 (7)	0.0384 (8)	0.0205 (5)	0.0064 (6)	0.0036 (4)	−0.0035 (5)
O5	0.0549 (9)	0.0469 (9)	0.0213 (5)	0.0213 (8)	0.0010 (5)	−0.0017 (5)

### Geometric parameters (Å, º)

**Table:**

C1—C9	1.373 (2)	C8—H8	0.9300
C1—C5	1.415 (2)	C9—H9	0.9300
C1—C2	1.416 (2)	C10—O3	1.201 (2)
C2—O1	1.343 (2)	C10—O2	1.325 (2)
C2—N1	1.372 (2)	C10—C11	1.506 (3)
C3—C4	1.367 (3)	C11—C12	1.522 (2)
C3—N1	1.403 (3)	C11—H11A	0.9700
C3—H3	0.9300	C11—H11B	0.9700
C4—C5	1.410 (3)	C12—C13	1.512 (3)
C4—H4	0.9300	C12—H12A	0.9700
C5—C6	1.418 (2)	C12—H12B	0.9700
C6—C7	1.368 (3)	C13—O5	1.248 (2)
C6—H6	0.9300	C13—O4	1.266 (2)
C7—C8	1.392 (3)	N1—H1	0.917 (10)
C7—H7	0.9300	O1—H1A	0.806 (10)
C8—C9	1.327 (2)	O2—H2A	0.833 (10)
			
C9—C1—C5	119.31 (14)	C8—C9—H9	119.1
C9—C1—C2	119.92 (15)	C1—C9—H9	119.1
C5—C1—C2	120.77 (15)	O3—C10—O2	119.75 (18)
O1—C2—N1	124.95 (15)	O3—C10—C11	124.01 (17)
O1—C2—C1	117.06 (15)	O2—C10—C11	116.24 (15)
N1—C2—C1	117.98 (16)	C10—C11—C12	113.75 (14)
C4—C3—N1	121.27 (18)	C10—C11—H11A	108.8
C4—C3—H3	119.4	C12—C11—H11A	108.8
N1—C3—H3	119.4	C10—C11—H11B	108.8
C3—C4—C5	119.22 (18)	C12—C11—H11B	108.8
C3—C4—H4	120.4	H11A—C11—H11B	107.7
C5—C4—H4	120.4	C13—C12—C11	114.45 (14)
C4—C5—C1	119.27 (16)	C13—C12—H12A	108.6
C4—C5—C6	122.75 (18)	C11—C12—H12A	108.6
C1—C5—C6	117.98 (16)	C13—C12—H12B	108.6
C7—C6—C5	120.24 (18)	C11—C12—H12B	108.6
C7—C6—H6	119.9	H12A—C12—H12B	107.6
C5—C6—H6	119.9	O5—C13—O4	122.73 (17)
C6—C7—C8	119.46 (16)	O5—C13—C12	120.36 (14)
C6—C7—H7	120.3	O4—C13—C12	116.91 (14)
C8—C7—H7	120.3	C2—N1—C3	121.47 (16)
C9—C8—C7	121.22 (18)	C2—N1—H1	119 (3)
C9—C8—H8	119.4	C3—N1—H1	119 (2)
C7—C8—H8	119.4	C2—O1—H1A	103 (2)
C8—C9—C1	121.79 (17)	C10—O2—H2A	122 (2)
			
C9—C1—C2—O1	−0.7 (2)	C5—C6—C7—C8	−1.0 (3)
C5—C1—C2—O1	178.07 (16)	C6—C7—C8—C9	0.3 (3)
C9—C1—C2—N1	179.79 (17)	C7—C8—C9—C1	0.3 (3)
C5—C1—C2—N1	−1.5 (3)	C5—C1—C9—C8	−0.3 (3)
N1—C3—C4—C5	−0.2 (3)	C2—C1—C9—C8	178.46 (18)
C3—C4—C5—C1	0.4 (3)	O3—C10—C11—C12	0.7 (3)
C3—C4—C5—C6	179.99 (19)	O2—C10—C11—C12	−178.94 (18)
C9—C1—C5—C4	179.17 (17)	C10—C11—C12—C13	178.42 (17)
C2—C1—C5—C4	0.4 (3)	C11—C12—C13—O5	−1.7 (3)
C9—C1—C5—C6	−0.4 (2)	C11—C12—C13—O4	177.79 (17)
C2—C1—C5—C6	−179.14 (17)	O1—C2—N1—C3	−177.81 (19)
C4—C5—C6—C7	−178.5 (2)	C1—C2—N1—C3	1.7 (3)
C1—C5—C6—C7	1.1 (3)	C4—C3—N1—C2	−0.9 (3)

### Hydrogen-bond geometry (Å, º)

**Table:**

*D*—H···*A*	*D*—H	H···*A*	*D*···*A*	*D*—H···*A*
N1—H1···O3^i^	0.92 (1)	2.48 (2)	3.255 (3)	142 (3)
O1—H1*A*···O4^ii^	0.81 (1)	1.91 (1)	2.705 (2)	170 (3)
O2—H2*A*···O5^iii^	0.83 (1)	1.77 (1)	2.591 (2)	169 (3)
C4—H4···O3^iv^	0.93	2.50	3.360 (3)	154
C8—H8···O5^v^	0.93	2.34	3.078 (3)	137
C9—H9···O4^v^	0.93	1.81	2.718 (2)	165

Symmetry codes: (i) −*x*, *y*−1/2, −*z*; (ii) −*x*, *y*+1/2, −*z*; (iii) −*x*, *y*+1/2, −*z*+1; (iv) *x*+1, *y*−1, *z*; (v) *x*, *y*+1, *z*.
